# Supplementary material for: Multimodal imaging and functional analysis of the chick NMDA retinal damage model
Source: PLoS One. 2021 Sep 7;16(9):e0257148. doi: 10.1371/journal.pone.0257148 (PMC8423281; doi:10.1371/journal.pone.0257148)
Supplement: S1 File — (ZIP) [file pone.0257148.s004.zip › SD-OCT/C200727/006/D28-Pwk5/C200727-006_OS_V_14X14_0_REGAVG0000137/Manual_PreDefineFiveByFiveGridRectangularRetina_1.pdf]

Five By Five Grid Template Report

**Patient ID**	C200727	**Name**	See InVivoVue
**Exam Date**	See InVivoVue	**Exam Time**	See InVivoVue
**Eye**	OS	**Averaged**	N/A
**Depth (mm)**	1.65	**Depth Samples**	1024
**Pitch (um/pix)**	35.000 x 35.000 x 1.608	**Refractive Index**	See InVivoVue
**Scan Type**	Rectangular 14.000mm x 14.000mm x 1.647mm , 400 x 400 x 1024		

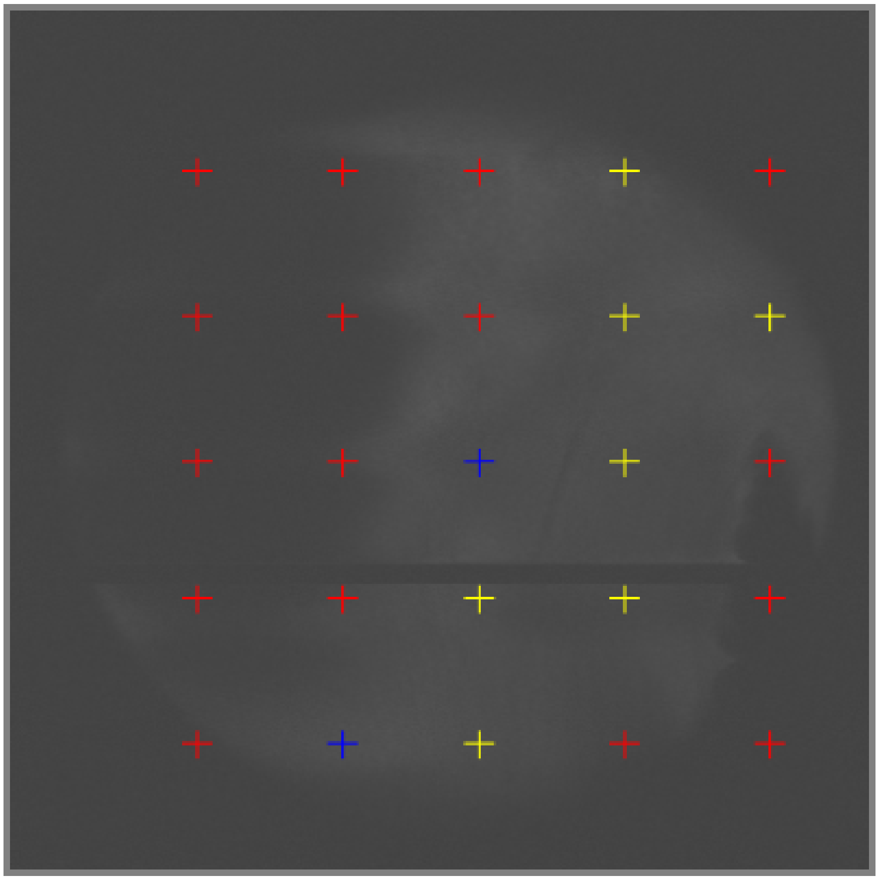

Thickness Metrics

Analysis	Average(mm)	Minimum(mm)	Maximum(mm)	STDEV(mm)
IRNFL - RPE	0.2434	0.2278	0.2590	0.0221
IRNFL - ORNFL	0.0459	0.0431	0.0487	0.0040
ORNFL - OPL	0.1180	0.1130	0.1229	0.0071
OPL - ETPRS	0.0633	0.0587	0.0680	0.0066

Q:\Bioptigen\Data\images\site3000254\image\2020.09.01\C200727-006_OS_V_14X14_0_REGAVG0000137.OCT 2/12/2021 2:51:21 PM
